# Disruption of brain rhythms in a pharmacological model of schizophrenia in male and female mice

**DOI:** 10.1186/s13293-025-00773-w

**Published:** 2025-11-07

**Authors:** Urte Jasinskyte, Robertas Guzulaitis

**Affiliations:** Life Sciences Center, Vilnius University, Vilnius, LT-10257 Lithuania

**Keywords:** Ketamine, Schizophrenia, Sex differences, Mice, Brain oscillations, Auditory steady-state response, Reliability

## Abstract

**Background:**

Brain oscillations are critical for neural communication and are increasingly recognized as sensitive biomarkers of neuropsychiatric dysfunction. Specifically, the auditory steady-state response (ASSR) has been identified as a reliable assessment of cortical entrainment and is a potential biomarker for the diagnosis or even prognosis of schizophrenia. Despite the growing awareness of sex as a biological variable, sex differences in oscillatory dynamics remain underexplored.

**Methods:**

Using electrophysiological recordings in mice, this study investigated sex differences in both spontaneous and evoked oscillatory brain activity under baseline conditions and following systemic NMDA receptor suppression via ketamine, a widely used pharmacological model of schizophrenia.

**Results:**

Although spontaneous oscillations across a wide range of frequency bands did not differ significantly between sexes, male mice exhibited greater gamma-band entrainment at 40 Hz ASSRs than females did. The administration of a subanaesthetic dose of ketamine consistently disrupted both spontaneous and gamma-band entrainment during ASSRs but without sex-specific effects. All measured oscillatory parameters showed high test‒retest reliability, thus indicating the robustness of the findings.

**Conclusions:**

Collectively, these results demonstrate sex-dependent differences in baseline cortical entrainment, highlighting the importance of including both sexes in preclinical research to fully elucidate the neurobiological mechanisms underlying brain oscillations and their implications in psychiatric disorders.

**Supplementary Information:**

The online version contains supplementary material available at 10.1186/s13293-025-00773-w.

## Abstract (plain language summary)

The brain communicates using electrical rhythms, similar to how musical beats guide the flow of a song. These rhythms, known as brain waves, are important for thinking, attention, and memory. Recent research suggests that brain rhythms might differ between males and females, yet many scientific studies typically include only male animals, leaving a critical gap in our understanding.

In this study, we explored whether brain rhythms differ between male and female mice, both naturally and after treatment with a drug called ketamine, which temporarily mimics symptoms similar to schizophrenia, a mental health condition. We found that natural, spontaneous brain rhythms were largely similar in male and female mice. However, when rhythmic sounds were played, male mice exhibited stronger brain rhythms, particularly in the gamma frequency range, important for information processing and attention. Furthermore, after ketamine treatment, both sexes showed similar disruptions in their brain rhythms, indicating that the drug affects male and female brains similarly.

This study emphasizes subtle yet meaningful differences in how male and female brains process sensory information. Including both sexes in neuroscience research is essential to fully understand normal brain functions and alterations during disorders, leading to better health outcomes for everyone.

**Supplementary Information:**

The online version contains supplementary material available at 10.1186/s13293-025-00773-w.

## Highlights

- Spontaneous cortical oscillations showed robust reliability and did not differ significantly between male and female mice.
- Male mice exhibited stronger 40 Hz auditory steady-state responses (ASSRs) compared to females.
- Systemic NMDA suppression with ketamine disrupted spontaneous oscillations, similarly affecting low-frequency decreases and high-frequency increases in both sexes.
- Ketamine administration significantly reduced 40 Hz ASSRs in male and female mice, with no sex-specific differences observed.

**Supplementary Information:**

The online version contains supplementary material available at 10.1186/s13293-025-00773-w.

## Introduction

Sex differences in brain function are increasingly recognized as crucial to understanding both normal and pathological neural processes. Oscillatory brain activity, particularly in the gamma frequency range, plays a fundamental role in cognitive functions, and disruptions of gamma oscillations have been implicated in a range of neuropsychiatric disorders, most notably schizophrenia [1–4]. However, sex-specific differences in these oscillatory dynamics remain poorly understood, particularly within preclinical models.

A robust tool for probing the oscillatory capabilities of neural circuits is the auditory steady-state response (ASSR), which reflects the ability of the brain to entrain to rhythmic auditory stimuli delivered at specific frequencies [5]. In particular, gamma-range ASSRs have emerged as promising biomarkers for neuropsychiatric conditions [6–12], most prominently schizophrenia [13–16], indicating impaired neural synchronization and entrainment. Gamma-band ASSRs critically depend on the interplay between excitatory and inhibitory neurotransmission [12], which is primarily mediated through parvalbumin-positive (PV) GABAergic interneurons and NMDA receptor function [17, 18]. Notably, both GABAergic signalling and NMDA receptor expression are influenced by sex hormones [19–21], suggesting that sex differences in oscillatory entrainment may be significant but are largely overlooked.

Indeed, accumulating evidence suggests that the neurobiological mechanisms underlying schizophrenia may be profoundly influenced by factors such as sex or hormonal status [22]. First, the incidence rates and symptom severity differ between men and women [23]. Second, women experience exacerbations of symptoms that are linked to their menstrual cycle [24]. Despite these observations, preclinical neuroscience research continues to utilize predominantly male animals [25, 26], driven by outdated assumptions and concerns regarding hormonal variability. Such sex bias risks obscuring meaningful biological differences and limits the translational relevance of animal models [27].

In the present study, we systematically investigated sex differences in spontaneous and evoked brain oscillations via chronic electrocorticogram (ECoG) recordings in awake, head-fixed mice. Furthermore, we tested whether subanaesthetic administration of ketamine, an NMDA receptor antagonist widely used to model schizophrenia-like states, differentially impacts these oscillations in male and female animals. By directly comparing oscillatory responses under both baseline and pharmacologically suppressed conditions, we aimed to identify sex-specific features of cortical network dynamics and emphasize the necessity of incorporating both sexes in preclinical research to fully elucidate the underlying neurobiological mechanisms relevant to psychiatric conditions.

## Materials and methods

### Animals

Male (n = 35) and female (n = 33) C57BL/6 mice, aged 9–17 weeks, were obtained from the Department of Biological Models, Vilnius University. The animals were housed in groups of up to six individuals per cage under a 12:12 light/dark cycle, with lights on at 7:00 am. All the experiments were conducted during the light phase.

### Surgical implantation of ECoG electrodes

Electrocorticogram (ECoG) electrodes and a metal head post were surgically implanted under sevoflurane anaesthesia (2–4% in 1 L/min O_2_, Sevohale, Chanelle Pharmaceuticals Manufacturing Ltd.). Body temperature was maintained at 36 °C using a temperature controller (ATC1000, WPI), and the depth of anaesthesia was monitored throughout the surgery. To prevent dehydration, eye ointment was applied, and the top of the head was disinfected with 70% ethanol and betadine. For local anaesthesia, lidocaine (Lidor, Richter Pharma) was injected subcutaneously at the incision site. When the required depth of anaesthesia was achieved, the scalp was incised, the skull was cleaned with a scalpel blade and 3% hydrogen peroxide, and marked for electrode implantation. Burr holes were mechanically drilled in the skull, and stainless-steel screw electrodes (2 mm length, 1 mm diameter) were implanted above the cerebral cortex in the following areas (from bregma): the auditory cortex (A1; AP −2.5 mm, ML 4.5 mm); the visual cortex (served as a reference (Ref); AP −3 mm, ML 2.5 mm); and above the cerebellum (served as ground; AP −6 mm, ML 1.5 mm). The electrodes and a metal head post (2 × 1 × 0.1 cm) were secured to the skull with dental cement (DMP Dental Industry). Postoperative care included intraperitoneal injections of the antibiotic enrofloxacin (5 mg/kg; Enroxil, KRKA) and the analgesic carprofen (4 mg/kg; Rycarfa, KRKA), administered immediately after surgery and once daily for three consecutive days. The animals were allowed at least one week of recovery before behavioural habituation.

### Habituation to the behavioural setup

The mice gradually habituated to head restraint while placing them into a plastic tube to minimize body movements. Habituation procedure took five days. Initially, brief handling and short periods (up to 2 min) of head restraint were employed, with the duration of fixation progressively increasing. The mice subsequently underwent four acclimation sessions in the recording plastic tube, with head fixation incrementally extended to 15 min per session. Auditory stimuli were introduced during the final four habituation sessions to acclimate the mice to the recording conditions.

### Drugs

Ketamine (Ketamidor, Richter Pharma) was dissolved in 0.9% saline solution (2 mg/ml). The mice received a subanaesthetic dose of ketamine (20 mg/kg, i.p.) or an equivalent volume of saline (control) 5 min before ECoG recordings.

### Auditory stimulation protocol

Auditory steady-state responses (ASSRs) were elicited via 2-ms white noise stimuli (clicks) generated by an Arduino Uno microcontroller triggered via a digitizer (Digidata 1440 A, Molecular Devices). Auditory stimuli were delivered in trains at 10, 20, 40, and 80 Hz, with each train lasting 1 s, separated by 1-s inter-stimulus intervals (ISIs). Each stimulation sequence was repeated 100 times per recording session at a consistent sound level (70 dBA at the animal’s head, measured via a DT-8851/8852 digital sound level meter).

### ECoG recording

ECoG recordings were conducted in awake, head-fixed mice within a low-light environment (5 lx). The signals were amplified via an Iso-DAM8A (WPI) differential amplifier (gain at 10^4^), bandpass (1–1000 Hz) and notch (50 Hz) filtered, sampled at 2 kHz (Digidata 1400 A, Molecular Devices), and digitally stored for subsequent analyses. To assess baseline brain oscillations, ECoGs were recorded daily for five consecutive days, which spans the average estrus cycle in mice [28]. To evaluate the effects of systemic NMDA suppression, ECoGs were recorded using 40 Hz stimulation paradigm 5 min after ketamine or saline administration, with drugs injected in a counterbalanced manner. NMDA suppression experiments were performed following 5-day baseline recording period. During ECoG recordings, mice were head-restrained using an implanted metal head post and placed in a plastic tube to suppress movement.

### Data analysis

Custom Python scripts (version 3.11) incorporating the MNE package (version 1.5.1) were used for analyses. Spontaneous brain activity was quantified via the multitaper method for power spectral density (PSD) analyses, specifically during epochs without stimulation (0.2–0.9 s prestimulus onset). The average PSDs were computed, and the frequency bands were defined as delta (2–4 Hz), theta (4–8 Hz), alpha (8–12 Hz), beta (12–30 Hz), low gamma (30–45 Hz), and high gamma (45–90 Hz).

For ASSR assessment, Morlet wavelet time‒frequency analysis was used to quantify the phase-locking factor (PLF), induced and evoked oscillatory responses. Induced power was computed as the power ratio (stimulation vs. baseline) and averaged across trials. Evoked power was assessed by first averaging ECoG trials and then performing time‒frequency analysis. Parameters were extracted specifically from auditory stimulation (0.2–0.9 s poststimulus onset) and baseline periods (0.1–0.5 s prestimulus onset) and averaged within frequency ranges (8–12, 18–22, 38–42, and 78–82 Hz).

The impact of KET was quantified by subtracting parameters obtained after the administration of KET from parameters obtained after the administration of SAL within individual animals.

### Statistical analysis

Statistical analyses were performed in Prism (GraphPad, version 8.0.2). Data normality was assessed via the Shapiro‒Wilk test. Paired comparisons employed paired t tests or Wilcoxon matched-pairs tests. Unpaired comparisons utilized unpaired t tests or Mann–Whitney U tests. The reliability of the measurements was assessed via intraclass correlation coefficients (ICCs, two-way mixed-effects model, absolute agreement), computed in RStudio (R, version 4.2.2), and interpreted as follows: poor (< 0.5), moderate (0.5–0.75), good (0.75–0.90), and excellent (> 0.90) reliability [29]. The data are presented as the means ± SDs.

## Results

### Brain oscillations in male and female mice

**Fig. 1:**
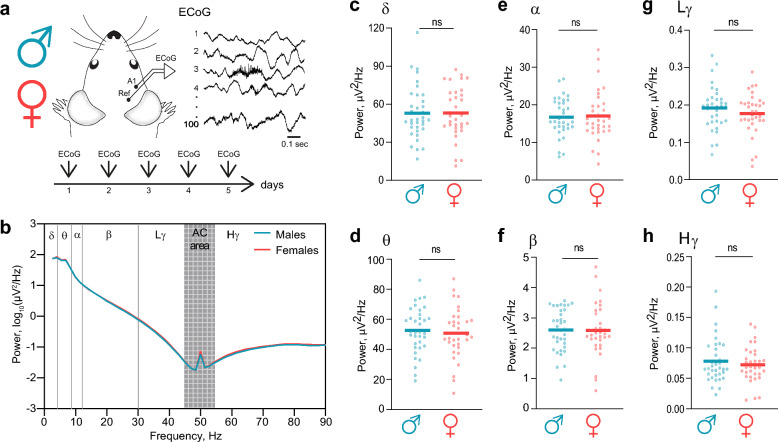
Spontaneous brain activity is comparable between male and female mice across a broad frequency range **a** Brain oscillations were recorded from the auditory cortex (A1) via ECoG in awake male and female mice during five consecutive days. **b** Grand average power spectral density (PSD) plots of spontaneous brain activity in male (cyan) and female (red) mice. Frequency bands were analysed: delta (δ, 2–4 Hz), theta (θ, 4–8 Hz), alpha (α, 8–12 Hz), beta (β, 12–30 Hz), low gamma (Lγ, 30–45 Hz), and high gamma (Hγ, 55–90 Hz). The AC band (45–55 Hz) was excluded from the analysis. **c‒h** Comparison of the power across frequency bands between male and female mice (δ: P = 0.99; θ: P = 0.6; α: P = 0.83; β: P = 0.95; Lγ: P = 0.29; Hγ: P = 0.93). Statistical comparisons were performed via the unpaired t test or the Mann‒Whitney U test. Significance notation: ns, non-significant (P > 0.05). Sample size: males, n = 35; females, n = 33

Brain oscillations in mice were assessed via two complementary approaches: spontaneous oscillations (recorded in the absence of external stimulation) and auditory steady-state responses (ASSRs), which evaluate network entrainment via rhythmic auditory stimulation. Recordings were conducted over five consecutive days, matching the typical duration of the mouse estrus cycle [28]. To characterize spontaneous brain oscillations, ECoG recordings were obtained from the auditory cortex (A1, Fig. 1a), and power spectral density (PSD) was computed (Fig. 1b). The PSD was analysed within standard frequency bands, including delta (δ, 2–4 Hz), theta (θ, 4–8 Hz), alpha (α, 8–12 Hz), beta (β, 12–30 Hz), low gamma (Lγ, 30–45 Hz), and high gamma (Hγ, 45–90 Hz) bands. The reliability of these spontaneous oscillations, recorded repeatedly over five consecutive days, was high, with intraclass correlation coefficients (ICCs) indicating good to excellent reliability across all frequency bands (Table 1). The average PSD profiles appeared to be very similar between male (cyan, n = 35) and female (red, n = 33) mice across all frequency ranges examined (Fig. 1b). Statistical comparisons confirmed the absence of significant sex differences across frequency bands, including delta (δ, Fig. 1c; P = 0.99), theta (θ, Fig. 1d; P = 0.6), alpha (α, Fig. 1e; P = 0.83), beta (β, Fig. 1f; P = 0.95), low gamma (Lγ, Fig. 1g; P = 0.29), and high gamma (Hγ, Fig. 1h; P = 0.93) bands. Consequently, spontaneous cortical oscillations were found to be reliably reproducible across days and did not differ significantly between sexes in any assessed frequency range.

**Table 1:** Intraclass correlation coefficients (ICCs) of spontaneous power at different frequency ranges for five consecutive days

**Oscillation band (Hz)**	**Sex**	**F-Test**	**ICC value**	**95 % CI**
Delta (2–4)	Males	30.8	0.85	0.77<ICC<0.91
	Females	15.4	0.74	0.62<ICC<0.84
Theta (4–8)	Males	41.9	0.89	0.83<ICC<0.94
	Females	65.1	0.93	0.89<ICC<0.96
Alpha (8–12)	Males	29.5	0.85	0.77<ICC<0.91
	Females	59.7	0.92	0.88<ICC<0.96
Beta (12–30)	Males	52.1	0.91	0.86<ICC<0.95
	Females	70.2	0.93	0.90<ICC<0.96
Low gamma (30–45)	Males	86	0.94	0.90<ICC<0.97
	Females	99	0.95	0.92<ICC<0.97
High gamma (55–90)	Males	29.4	0.85	0.77<ICC<0.91
	Females	29.9	0.85	0.77<ICC<0.92

**Fig. 2:**
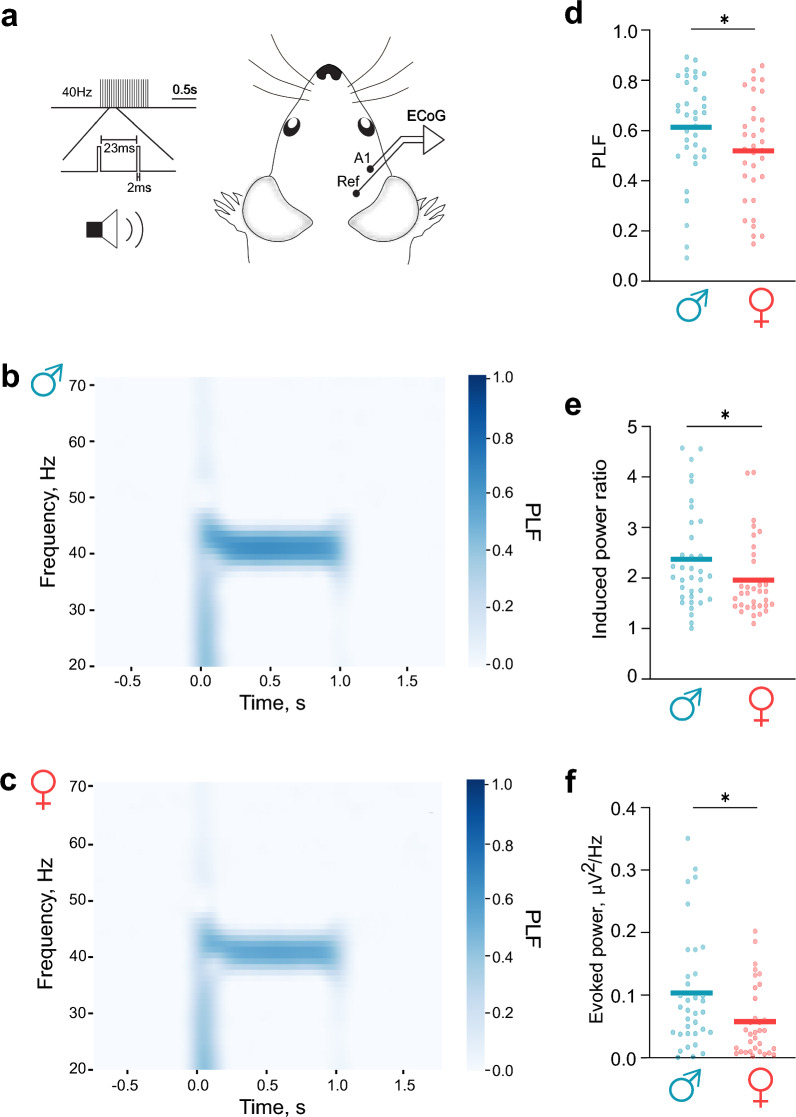
Male mice exhibit enhanced 40 Hz auditory steady-state responses (ASSRs) **a** ECoG activity was recorded from the auditory cortex (A1) during the presentation of rhythmic white noise stimuli (clicks) at 40 Hz. **b, c** Time‒frequency plots of ASSRs quantified by the phase-locking factor (PLF) in male (**b**) and female (**c**) mice. **d** Comparison of the PLF induced by 40 Hz stimulation in male (cyan) and female mice (red, P = 0.041). **e** Comparison of the induced power ratio at 40 Hz stimulation in male (cyan) and female mice (red, P = 0.039). **f** Comparison of evoked power at 40 Hz stimulation in male (cyan) and female mice (red, P = 0.023). Statistical comparisons were performed via the unpaired t test or the Mann‒Whitney U test. Significance notation: *, P < 0.05. Sample size: males, n = 35; females, n = 33

Despite the absence of sex differences in spontaneous oscillations, male and female mice brains may differ in their capacity to oscillate in response to rhythmic sensory stimulation, such as auditory stimulation generating ASSRs (Fig. 2a). To evaluate this possibility, ASSRs were recorded from male and female mice across five consecutive days. Auditory stimulation reliably enhanced oscillations at the frequency matching the stimulation frequency i.e. 40 Hz, as indicated by phase-locking factor (PLF) measurements for both male (Fig. 2b) and female (Fig. 2c) mice. Similarly to previous observations [30], PLF values increased systematically for 40 Hz and other stimulation frequencies across the five-day recording period, with the exception of 20 Hz (Supplementary Fig. 1, Table 2). Furthermore, the increase in PLFs, i.e. the difference in PLF between the fifth and the first recording day (Δ PLF), was similar for both sexes (Table 2). Notably, PLF during 40 Hz ASSR stimulation was significantly greater in males than in females (Fig. 2d; P = 0.041), whereas no significant sex differences emerged at the other tested frequencies (Supplementary Fig. 2a, Table 3). Similarly, the induced power ratio, calculated as power during auditory stimulation relative to the baseline, was significantly greater in males than in females at 40 Hz ASSRs (Fig. 2e; P = 0.039) but not at other frequencies (Supplementary Fig. 2b, Table 3). Furthermore, the evoked power, calculated by averaging power across recording episodes during auditory stimulation, was significantly greater in males at 40 Hz stimulation (Fig. 2f; P = 0.023), with no significant differences found at other frequencies (Supplementary Fig. 2c, Table 3). The reliability of these ASSR parameters, as determined via the ICC across the five consecutive recording days, demonstrated good to excellent reliability in all cases (Table 4). Auditory stimulation induces neural entrainment not only at the stimulation frequency, but also at its higher multiples called harmonic responses (Supplementary Fig. 3), which are believed could also serve as a reliable biomarker [31]. The first harmonics of different stimulation frequencies showed high reliability, as determined via the ICC analysis (Table 5) and these harmonics were similar in male and female mice (Supplementary Fig. 3). The findings reveal robust and reliable sex-dependent differences, specifically at 40 Hz cortical entrainment, highlighting enhanced gamma-frequency synchronization in male mice compared with females, despite similar spontaneous oscillatory dynamics.

**Table 2:** Changes of ASSRs parameters across five consecutive days at different stimulation frequencies

**ASSRs parameter**	**Stimulation frequency**	**Day**	**Sex**	**Mean ± SD**	**Test**
PLF	10 Hz	1	Males	0.27 ± 0.10	**P = 5 × 10**^**−4**^; Wilcoxon
		5		0.32 ± 0.13	
		1	Females	0.29 ± 0.12	**P = 2 × 10**^**−3**^; Wilcoxon
		5		0.32 ± 0.12	
	20 Hz	1	Males	0.48 ± 0.17	P = 0.17; paired t
		5		0.50 ± 0.16	
		1	Females	0.48 ± 0.15	P = 0.21; paired t
		5		0.50 ± 0.15	
	40 Hz	1	Males	0.59 ± 0.20	**P = 4 × 10**^**−3**^; Wilcoxon
		5		0.64 ± 0.21	
		1	Females	0.47 ± 0.20	**P = 4 × 10**^**−3**^; Wilcoxon
		5		0.54 ± 0.23	
	80 Hz	1	Males	0.28 ± 0.12	**P = 1 × 10**^**−5**^; paired t
		5		0.37 ± 0.16	
		1	Females	0.33 ± 0.14	**P = 2 × 10**^**−3**^; paired t
		5		0.38 ± 0.18	
Δ PLF	10 Hz	5-1	Males	0.05 ± 0.07	P = 0.39; unpaired t
			Females	0.03 ± 0.05	
	20 Hz	5-1	Males	0.02 ± 0.09	P = 0.86, unpaired t
			Females	0.02 ± 0.08	
	40 Hz	5-1	Males	0.05 ± 0.1	P = 0.67, unpaired t
			Females	0.06 ± 0.11	
	80 Hz	5-1	Males	0.08 ± 0.1	P = 0.14, unpaired t
			Females	0.05 ± 0.1	

*P* < 0.05 are shown in bold

**Table 3:** Parameters of ASSRs assessed at different stimulation frequencies for five consecutive days

**ASSRs parameter**	**Stimulation frequency**	**Sex**	**Mean ± SD**	**Test**
PLF	10 Hz	Males	0.30 ± 0.12	P = 0.647; unpaired t
		Females	0.31 ± 0.12	
	20 Hz	Males	0.49 ± 0.16	P = 0.877; unpaired t
		Females	0.49 ± 0.14	
	40 Hz	Males	0.61 ± 0.20	**P =** **0.041**; Mann-Whitney U
		Females	0.52 ± 0.21	
	80 Hz	Males	0.32 ± 0.13	P = 0.540; Mann-Whitney U
		Females	0.36 ± 0.16	
Induced power ratio	10 Hz	Males	0.99 ± 0.17	P = 0.223; unpaired t
		Females	0.94 ± 0.13	
	20 Hz	Males	1.46 ± 0.39	P = 0.306; Mann-Whitney U
		Females	1.35 ± 0.30	
	40 Hz	Males	2.37 ± 1.00	**P =** **0.039**; Mann-Whitney U
		Females	1.96 ± 0.77	
	80 Hz	Males	2.16 ± 1.35	P = 0.317; Mann-Whitney U
		Females	2.30 ± 1.26	
Evoked power	10 Hz	Males	2.14 ± 1.74	P = 0.826; Mann-Whitney U
		Females	2.13 ± 1.65	
	20 Hz	Males	1.16 ± 1.06	P = 0.600; Mann-Whitney U
		Females	0.89 ± 0.63	
	40 Hz	Males	0.10 ± 0.09	**P =** **0.023**; Mann-Whitney U
		Females	0.06 ± 0.06	
	80 Hz	Males	0.02 ± 0.03	P = 0.874; Mann-Whitney U
		Females	0.03 ± 0.04	

*P* < 0.05 are shown in bold

**Table 4:** Intraclass correlation coefficients (ICCs) of ASSRs at different stimulation frequencies for five consecutive days

**ASSRs parameter**	**Stimulation frequency**	**Sex**	**F-Test**	**ICC value**	**95 % CI**
PLF	10 Hz	Males	51.6	0.89	0.83<ICC<0.94
		Females	64.5	0.92	0.87<ICC<0.96
	20 Hz	Males	65.2	0.93	0.89<ICC<0.96
		Females	47.3	0.9	0.85<ICC<0.94
	40 Hz	Males	99.2	0.94	0.91<ICC<0.97
		Females	66.1	0.91	0.86<ICC<0.95
	80 Hz	Males	24.7	0.79	0.66<ICC<0.88
		Females	37.4	0.87	0.79<ICC<0.92
Induced power ratio	10 Hz	Males	16.5	0.74	0.63<ICC<0.84
		Females	11.7	0.68	0.55<ICC<0.80
	20 Hz	Males	23.3	0.82	0.72<ICC<0.89
		Females	18.1	0.78	0.67<ICC<0.87
	40 Hz	Males	51.8	0.9	0.85<ICC<0.94
		Females	46.6	0.89	0.83<ICC<0.94
	80 Hz	Males	20	0.79	0.69<ICC<0.87
		Females	12.5	0.69	0.55<ICC<0.81
Evoked power	10 Hz	Males	36.3	0.85	0.76<ICC<0.91
		Females	41.5	0.88	0.82<ICC<0.93
	20 Hz	Males	54.6	0.92	0.87<ICC<0.95
		Females	33.1	0.87	0.79<ICC<0.92
	40 Hz	Males	65.3	0.91	0.85<ICC<0.95
		Females	51.9	0.9	0.83<ICC<0.94
	80 Hz	Males	14.6	0.71	0.59<ICC<0.82
		Females	66.6	0.93	0.88<ICC<0.96

**Table 5:** Intraclass correlation coefficients (ICCs) of the first harmonic of PLFs at different stimulation frequencies for five consecutive days

**Stimulation frequency**	**Harmonic**	**Sex**	**F-Test**	**ICC value**	**95 % CI**
10 Hz	20 Hz	Males	21.9	0.79	0.69<ICC<0.88
		Females	27.5	0.83	0.74<ICC<0.90
20 Hz	40 Hz	Males	63	0.91	0.85<ICC<0.95
		Females	45.4	0.84	0.68<ICC<0.92
40 Hz	80 Hz	Males	18.3	0.74	0.60<ICC<0.84
		Females	15.1	0.71	0.58<ICC<0.83

### Effects of ketamine administration on brain oscillations in male and female mice

**Fig. 3:**
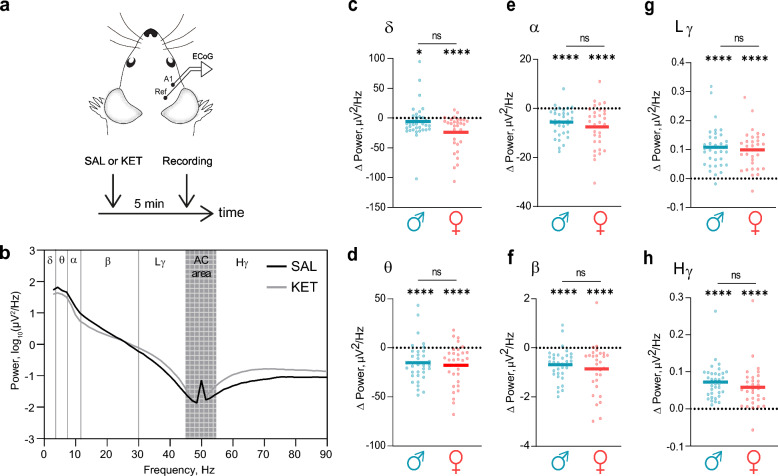
Systemic ketamine administration similarly altered spontaneous oscillations in male and female mice **a** ECoG activity was recorded from the auditory cortex (A1) 5 min after the administration of ketamine (KET) or saline (SAL). **b** Power spectral density (PSD) plots of spontaneous brain activity in all mice after saline (SAL, black) and ketamine (KET, grey) administration. Frequency bands analysed: delta (δ, 2–4 Hz), theta (θ, 4–8 Hz), alpha (α, 8–12 Hz), beta (β, 12–30 Hz), low gamma (Lγ, 30–45 Hz), and high gamma (Hγ, 55–90 Hz) bands. The AC band (45–55 Hz) was excluded from the analysis. **c‒f** Comparisons of KET-induced decreases in power at delta (δ, **c**), theta (θ, **d**), alpha (α, **e**) and beta (β, **f**) frequencies between male and female mice. **g, h** Comparisons of KET-induced increases in power at low gamma (Lγ, **g**) and high gamma (Hγ, **h**) frequency ranges between male and female mice. Statistical comparisons of power values were performed via the paired t test or the Wilcoxon test; group comparisons of Δ Power were evaluated using unpaired t test or the Mann‒Whitney U test. Significance notation: ns, non-significant (P > 0.05); *, P < 0.05; ****, P < 0.0001. Sample size: males, n = 35; females, n = 31

Emerging evidence suggests that sex influences the pathophysiology of schizophrenia [32]. Although schizophrenia has been shown to alter brain oscillations across multiple frequency bands [33], potential sex differences remain understudied. To address this gap, we employed a pharmacological model using ketamine (KET), an NMDA receptor antagonist known to replicate schizophrenia-like pathophysiology in rodents [34–36]. To assess the impact of KET, ECoG recordings were performed following the administration of either KET or saline (SAL; control) (Fig. 3a). To evaluate changes in spontaneous oscillations, the PSDs were calculated after KET and SAL administration (Fig. 3b). Next, Δ Power was calculated by subtracting power values obtained after saline administration from power values after ketamine administration for each frequency band (Δ Power = Power_KET – Power_SAL). KET administration resulted in a reduction in low-frequency oscillations in both male and female mice (Fig. 3b, Table 6). However, no statistically significant sex differences were observed in these changes across any low-frequency bands, including delta (Fig. 3c; δ, P = 0.14), theta (Fig. 3d; θ, P = 0.61), alpha (Fig. 3e; α, P = 0.28), and beta (Fig. 3f; β, P = 0.71) bands. Similarly, the administration of KET increased power in high-frequency oscillations (low and high gamma) in both sexes (Fig. 3b, Table 6), but again, there were no significant differences between males and females (Fig. 3g, h; Lγ, P = 0.78; Hγ, P = 0.11). Together, these findings indicate that ketamine-induced alterations in spontaneous brain oscillations are sex-independent, affecting both low- and high-frequency bands similarly in male and female mice.

**Table 6:** Spontaneous brain activity assessed for males and females at a different frequency ranges after the administration of saline and ketamine

**Oscillation band (Hz)**	**Group**	**Sex**	**Power, μV**^**2**^**/Hz, mean ± SD**	**Test**
Delta (2–4)	Saline	Males	48.67 ± 22.56	**P =** **0.015**, Wilcoxon
	Ketamine		43.17 ± 25.42	
	Saline	Females	60.01 ± 33.73	**P = 1 × 10**^**−5**^, paired t
	Ketamine		35.88 ± 18.67	
Theta (4–8)	Saline	Males	54.30 ± 19.21	**P = 5 × 10**^**−5**^, Wilcoxon
	Ketamine		39.07 ± 18.21	
	Saline	Females	54.93 ± 25.26	**P = 6 × 10**^**−5**^, paired t
	Ketamine		37.12 ± 19.36	
Alpha (8–12)	Saline	Males	17.00 ± 5.91	**P = 1 × 10**^**−6**^, paired t
	Ketamine		11.44 ± 4.67	
	Saline	Females	17.33 ± 9.78	**P = 5 × 10**^**−5**^, paired t
	Ketamine		9.81 ± 5.80	
Beta (12–30)	Saline	Males	2.77 ± 0.90	**P = 2 × 10**^**−7**^, paired t
	Ketamine		2.09 ± 0.77	
	Saline	Females	2.75 ± 1.36	**P = 3 × 10**^**−5**^, paired t
	Ketamine		1.89 ± 0.94	
Low gamma (30–45)	Saline	Males	0.20 ± 0.08	**P = 2 × 10**^**−10**^, paired t
	Ketamine		0.31 ± 0.13	
	Saline	Females	0.17 ± 0.08	**P = 3 × 10**^**−9**^, paired t
	Ketamine		0.27 ± 0.13	
High gamma (55–90)	Saline	Males	0.08 ± 0.03	**P = 6 × 10**^**−11**^, Wilcoxon
	Ketamine		0.15 ± 0.07	
	Saline	Females	0.07 ± 0.03	**P = 2 × 10**^**−7**^, Wilcoxon
	Ketamine		0.13 ± 0.08	

*P* < 0.05 are shown in bold

**Fig. 4:**
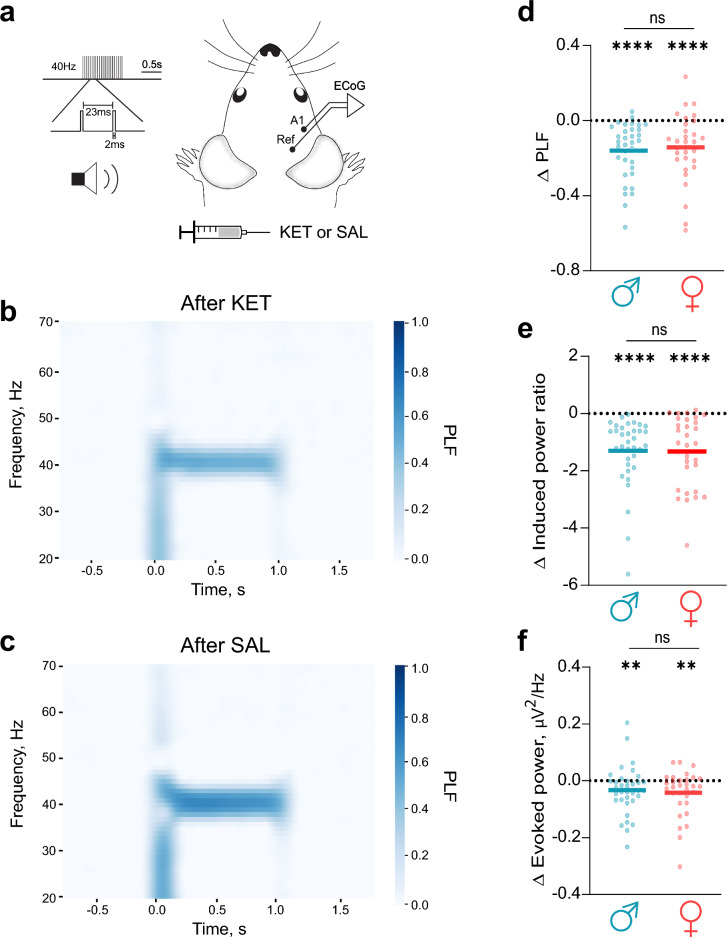
Ketamine similarly disrupts 40 Hz ASSRs in male and female mice **a** ECoG activity was recorded from the auditory cortex (A1) during 40 Hz click presentation after ketamine (KET) or saline (SAL) administration. **b, c** Time‒frequency plots of the grand average phase-locking factor (PLF) after KET (**b**) and SAL (**c**) administration (n = 66). **d‒f** Comparisons of KET-induced disruption of ASSRs between males (cyan) and females (red) evaluated as change in PLF (Δ PLF, **d**), induced power (Δ Induced power ratio, **e**), and evoked power (Δ Evoked power, **f**) after KET administration compared to SAL administration. Statistical comparisons of change in ASSR values after KET were performed via the paired t test or the Wilcoxon test; group comparisons of KET induced changes (Δ) were evaluated using the unpaired t test or the Mann‒Whitney U test. Significance notation: ns, non-significant (P > 0.05); **, P < 0.001; ****, P < 0.0001. Sample size: males, n = 35; females, n = 31

Given that alterations in gamma-band ASSRs have been consistently observed in schizophrenia [12] and in pharmacological NMDA-suppressed rodent models [37–39], we next assessed whether KET differentially impacts ASSRs between sexes. ASSRs were elicited via 40 Hz auditory stimulation following either KET or SAL administration in male and female mice (Fig. 4a). Qualitative time–frequency analysis demonstrated a notable reduction in the PLF following KET administration (Fig. 4b), compared with that following SAL administration (Fig. 4c). Statistical analyses confirmed that, compared with SAL, KET significantly reduced 40 Hz PLF (Fig. 4d), induced power (Fig. 4e), and evoked power (Fig. 4f) in both male and female mice (Table 7). However, no significant sex differences were observed in these ketamine-induced reductions (Fig. 4d-f; PLF, P = 0.76; induced power, P = 0.85; evoked power, P = 0.88). In contrast to effects on ASSRs, KET did not change the 80 Hz harmonic response during 40 Hz stimulation, in either male or female mice (Supplementary Fig. 4). Consequently, although ketamine robustly disrupted 40 Hz ASSRs, this disruption occurred similarly in males and females.

**Table 7:** Parameters of ASSRs assessed for males and females after the administration of saline and ketamine

**ASSRs parameter**	**Group**	**Sex**	**Mean ± SD**	**Paired wilcoxon test**
PLF	Saline	Males	0.67 ± 0.18	**P = 5 × 10**^**−9**^
	Ketamine		0.51 ± 0.24	
	Saline	Females	0.62 ± 0.22	**P = 4 × 10**^**−5**^
	Ketamine		0.48 ± 0.22	
Induced power ratio	Saline	Males	3.06 ± 1.86	**P = 6 × 10**^**−11**^
	Ketamine		1.76 ± 1.03	
	Saline	Females	2.86 ± 1.66	**P = 5 × 10**^**−8**^
	Ketamine		1.54 ± 0.60	
Evoked power	Saline	Males	0.16 ± 0.16	**P = 0.005**
	Ketamine		0.13 ± 0.17	
	Saline	Females	0.13 ± 0.15	**P = 0.005**
	Ketamine		0.09 ± 0.12	

*P* < 0.05 are shown in bold

## Discussion

The present study systematically investigated sex differences in spontaneous and evoked brain oscillations in mice, both under baseline conditions and following systemic NMDA receptor suppression by ketamine (KET), a pharmacological model widely used to mimic schizophrenia-like states. Spontaneous oscillations exhibited remarkable stability over multiple days, demonstrating no substantial sex differences across the entire frequency spectrum. In contrast, male mice had stronger ASSRs at 40 Hz, as reflected by higher PLF, induced and evoked power, suggesting increased gamma-range entrainment in the auditory system. Following KET administration, spontaneous oscillations underwent significant alterations, with decreases in low-frequency bands and increases in gamma frequencies, equivalently affecting both male and female mice. Similarly, KET robustly disrupted 40 Hz ASSRs, reducing neural entrainment uniformly in both sexes. Consequently, our findings highlight subtle yet functionally relevant sex differences in cortical network dynamics, particularly those involving gamma-frequency entrainment.

Sex differences in brain function have been increasingly attributed to complex interactions among hormonal, genetic, and neurodevelopmental factors [40–42]. In this study, the most pronounced sex difference emerged at 40 Hz ASSRs, with stronger gamma entrainment observed in male mice. Oestrogen is known to modulate key components underpinning gamma oscillations, including GABAergic neurotransmission [43] and NMDA receptor activity [20]. Notably, prior research has demonstrated that changes in 40 Hz ASSRs are linked to estrogen fluctuations across the estrus cycle [30]. However, these changes were modest [30], which may explain the similar stability of ASSRs across multiple recording days, as reflected by ICC, in both male and female mice. This suggests that other variables, such as stress or arousal, may also contribute to the sex differences observed in this study. Although hormonal modulation presents a plausible biological explanation for the observed sex-specific gamma entrainment, targeted studies explicitly manipulating estrogen levels are needed to confirm causality and the underlying mechanisms.

In humans, the auditory system also exhibits sex differences and hormone dependence [44], although evidence specifically on ASSRs remains sparse. Similar to the variation during the estrus cycle in mice [30], ASSRs vary across the menstrual cycle in women [45]. Higher 40 Hz ASSRs have been reported in men compared with women, but only in left-handed populations [46], whereas lower ASSR thresholds have been observed in women [47]. Furthermore, some studies found no sex differences in ASSRs [48, 49], highlighting the need for further research into sex-specific auditory processing. While our findings demonstrate biological sex differences in mice, caution is warranted when translating these results to humans. In clinical populations, symptom expression and treatment outcomes are influenced not only by biological sex but also by gender as a social construct, which shapes lived experience [50, 51].

Although no significant sex differences were detected in spontaneous oscillations in this study, some human studies have reported varying results, with higher delta [52, 53], beta [52–54], and gamma [54, 55] values in females than in males. The evidence for alpha-band differences remains mixed, showing either greater power in males [53] or females [52, 55]. Furthermore, such differences may be contingent on behavioural states (e.g., eyes-open or eyes-closed) [55], thereby exacerbating the complexity of direct comparisons. In rodents, evidence for sex differences in spontaneous oscillations remains sparse, largely derived from in vitro experiments [56, 57], thus limiting translation to intact network dynamics in vivo. Here, the spontaneous activity was assessed during inter-stimulus intervals (ISIs), and these short ISIs may not fully capture resting-state dynamics. Factors such as arousal or expectancy can have an influence, and more extended rest periods may provide a clearer picture of spontaneous activity. This may partly explain the absence of sex differences in spontaneous oscillations. Encouragingly, increasing the inclusion of both sexes in neuroscience research [27, 51] should provide further clarity on potential sex-dependent patterns of spontaneous brain activity in future studies.

Spontaneous oscillations and ASSRs demonstrated robust reliability, as validated over consecutive recording sessions. Good reliability for spontaneous activity [58] and 40 Hz ASSRs [59, 60] has been established in human studies, although limited data exist for rodents. Prior rodent studies reported that 40 Hz ASSRs are reliable exclusively in females [30, 61]. The present study extends these findings, confirming the good reliability of spontaneous oscillations and ASSRs at multiple frequencies in both male and female mice, thus supporting the robustness and translational validity of these measures.

The use of systemic NMDA receptor antagonists is well established for modelling schizophrenia-like states in rodents [62–64]. In this study, the administration of KET significantly disrupted spontaneous oscillations, notably elevating high-frequency activity and reducing lower-frequency activity similarly in male and female mice. These findings align closely with prior studies demonstrating that gamma oscillation increases following NMDA receptor antagonism [37, 65–68]. Furthermore, the administration of KET robustly suppressed 40 Hz ASSRs in both male and female mice, paralleling decreases in neural entrainment that have been observed in schizophrenia patients [16, 69]. However, preclinical studies have yielded conflicting results regarding the effects of NMDA antagonists on gamma entrainment, with some reporting decreased [37–39, 65, 70] and others increased gamma entrainment [71–75]. This disparity suggests that gamma entrainment may be influenced by various factors that are not always controlled for in these studies or by differences in experimental design. For example, the ISIs used in ASSR studies can significantly influence measured responses. Using shorter ISIs reveals larger differences in ASSR parameters between healthy individuals and schizophrenia patients [76]. Thus, the choice of ISI may partly explain discrepancies in the literature regarding the effects of NMDA receptor antagonism on ASSRs. Additionally, increased stress levels during head-fixation could contribute to variability in responses. Although we habituated mice to head-fixation over multiple days prior to ECoG recordings, a procedure previously shown to substantially reduce stress levels [77], we did not systematically monitor behavioural, physiological, or hormonal stress indicators. Therefore, residual stress could influence brain oscillations in both baseline and ketamine conditions. Nevertheless, the present study demonstrated that NMDA suppression similarly altered ASSRs in both male and female mice, suggesting that the differences observed in previous studies regarding the direction of gamma entrainment changes are unlikely to be attributed to sex differences.

*Perspective and Significance*. This study contributes to a growing body of research emphasizing the need to consider sex as a biological variable in neuroscience. The present findings highlight the importance of sex-specific differences in auditory gamma entrainment, emphasising the intricate relationship between sex and cortical network dynamics. The robustness of both spontaneous and evoked oscillations across sexes strengthens their utility as translational biomarkers in preclinical models. In future studies, the integration of hormonal profiling or manipulations, such as estrogen modulation, will be crucial to delineating the mechanistic basis of sex differences in neural oscillations. These efforts may pave the way for more sex-sensitive diagnostics and therapeutic interventions in disorders characterized by oscillatory dysfunction, such as schizophrenia.

## Conclusions

In summary, this study demonstrates that while spontaneous brain oscillations are stable and largely similar between male and female mice, evoked auditory gamma oscillations (40 Hz ASSRs) exhibit subtle yet significant sex differences, with males showing stronger entrainment. Despite this baseline divergence, NMDA receptor suppression by ketamine disrupted both spontaneous and evoked activity similarly in both sexes. These findings support the robustness and translational relevance of electrophysiological biomarkers in rodent models and highlight the importance of including both sexes in neuroscience research to capture the full spectrum of biological variability.

## Supplementary Information

Supplementary Material 1: Figure 1. PLF of ASSRs changes similarly across 5-day baseline recordings in male and female mice. a-d Phase-locking factors (PLFs) at 10 (a), 20 (b), 40 (c), and 80 Hz (d) stimulation frequencies recorded over five consecutive days in male (cyan) and female (red) mice (left). Changes in PLF over days (Δ PLF) were assessed as the difference between PLF values on Day 5 and Day 1 of ECoG recordings (on the right). Statistical comparisons of PLF values across days were performed via the paired t test or the Wilcoxon test; group comparisons of Δ PLF were evaluated using the unpaired t test or the Mann‒Whitney U test. Significance notation: ns, non-significant (P > 0.05). Sample size: males, n = 35; females, n = 33.

Supplementary Material 2: Figure 2. Similar ASSRs induced by 10, 20 and 80 Hz stimulation in male and female mice. a Phase-locking factors (PLFs) of ASSRs evoked by 10, 20, and 80 Hz stimulation frequencies in male (cyan) and female (red) mice. b Induced power ratios of ASSRs evoked by 10, 20, and 80 Hz stimulation frequencies in male (cyan) and female (red) mice. c Evoked power of ASSRs evoked by 10, 20, and 80 H z stimulation frequencies in male (cyan) and female (red) mice. Statistical comparisons were performed via the unpaired t test or the Mann‒Whitney U test. Significance notation: ns, non-significant (P > 0.05). Sample size: males, n = 35; females, n = 33.

Supplementary Material 3:Figure 3. Comparable harmonic response of ASSRs in male and female mice. a-c First harmonic responses of ASSRs at 10 (a), 20 (b) and 40 Hz (c) stimulation frequencies in male and female mice. Statistical comparisons were performed via the unpaired t test or the Mann-Whitney test. Significance notation: ns, non-significant (P > 0.05). Sample size: males, n = 35; females, n = 33.

Supplementary Material 4: Figure 4. Ketamine does not affect the 80 Hz harmonic response during 40 Hz stimulation in male and female mice. a, b Time‒frequency plots of the grand average phase-locking factor (PLF) following ketamine (KET, a) and saline (SAL, b) administration (n = 66). The black rectangles show the area of the first harmonics analysed. c, d PLF at the 80 Hz harmonic response during 40 Hz stimulation in male (c) and female (d) mice after KET and SAL. e Ketamine effects on the 80 Hz harmonic were calculated as Δ PLF (PLF_KET – PLF_SAL). Statistical comparisons of PLF values were performed via the paired t test or the Wilcoxon test; group comparisons of Δ PLF were evaluated using unpaired t test. Significance notation: ns, non-significant (P > 0.05). Sample size: males, n = 35; females, n = 31.

## Data Availability

The datasets used and/or analysed during the current study are available from the corresponding author on reasonable request.
