# Supplementary figures and images for: Disruption of brain rhythms in a pharmacological model of schizophrenia in male and female mice
Source: Biol Sex Differ. 2025 Nov 7;16:94. doi: 10.1186/s13293-025-00773-w (PMC12595673; doi:10.1186/s13293-025-00773-w)

**a** 10 Hz

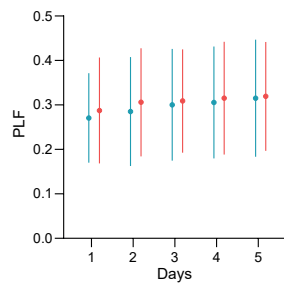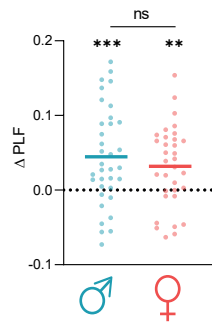

**c** 40 Hz

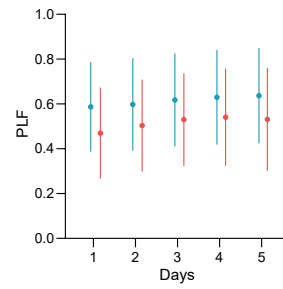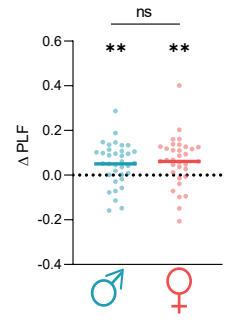

**b** 20 Hz

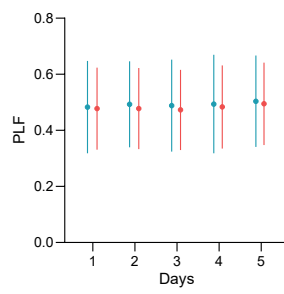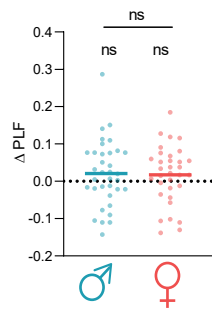

**d** 80 Hz

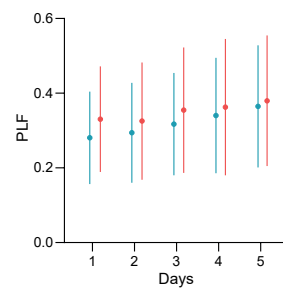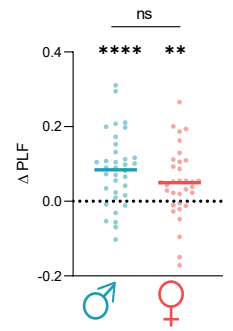

Supplement: Supplementary file 1 — Supplementary Material 1: Figure 1. PLF of ASSRs changes similarly across 5-day baseline recordings in male and female mice. a-d Phase-locking factors (PLFs) at 10 (a), 20 (b), 40 (c), and 80 Hz (d) stimulation frequencies recorded over five consecutive days in male (cyan) and female (red) mice (left). Changes in PLF over days (Δ PLF) were assessed as the difference between PLF values on Day 5 and Day 1 of ECoG recordings (on the right). Statistical comparisons of PLF values across days were performed via the paired t test or the Wilcoxon test; group comparisons of Δ PLF were evaluated using the unpaired t test or the Mann‒Whitney U test. Significance notation: ns, non-significant (P > 0.05). Sample size: males, n = 35; females, n = 33. [file 13293_2025_773_MOESM1_ESM.pdf]

**a** 10 Hz

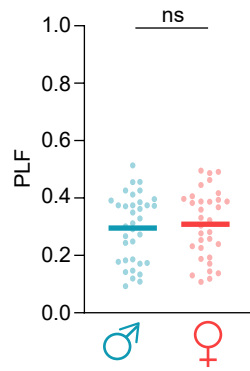

20 Hz

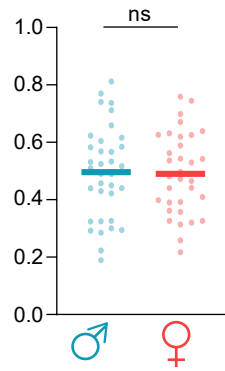

80 Hz

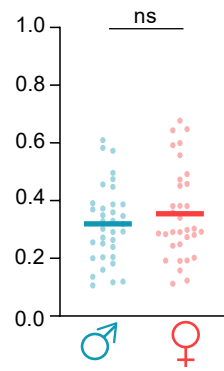

**b** 10 Hz

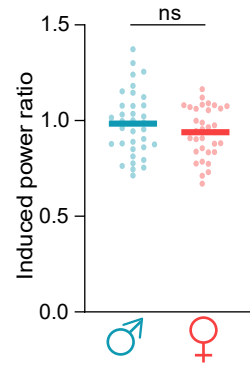

20 Hz

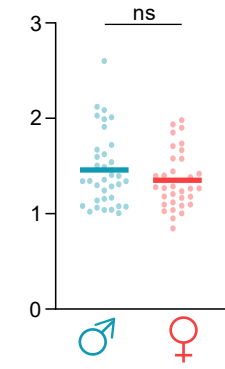

80 Hz

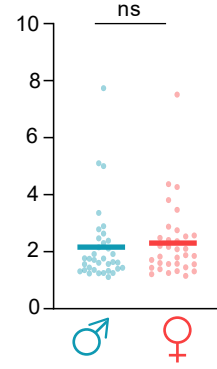

**c** 10 Hz

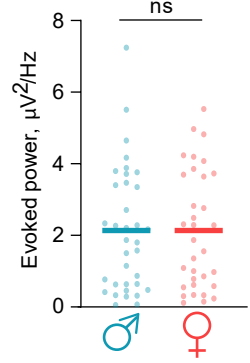

20 Hz

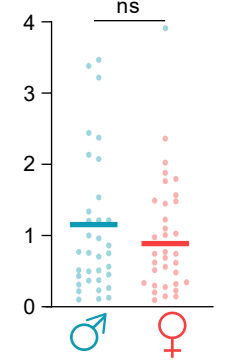

80 Hz

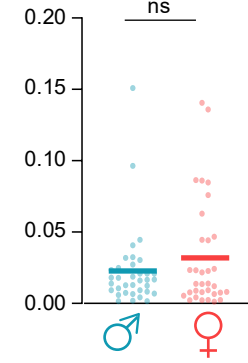

Supplement: Supplementary file 2 — Supplementary Material 2: Figure 2. Similar ASSRs induced by 10, 20 and 80 Hz stimulation in male and female mice. a Phase-locking factors (PLFs) of ASSRs evoked by 10, 20, and 80 Hz stimulation frequencies in male (cyan) and female (red) mice. b Induced power ratios of ASSRs evoked by 10, 20, and 80 Hz stimulation frequencies in male (cyan) and female (red) mice. c Evoked power of ASSRs evoked by 10, 20, and 80 H z stimulation frequencies in male (cyan) and female (red) mice. Statistical comparisons were performed via the unpaired t test or the Mann‒Whitney U test. Significance notation: ns, non-significant (P > 0.05). Sample size: males, n = 35; females, n = 33. [file 13293_2025_773_MOESM2_ESM.pdf]

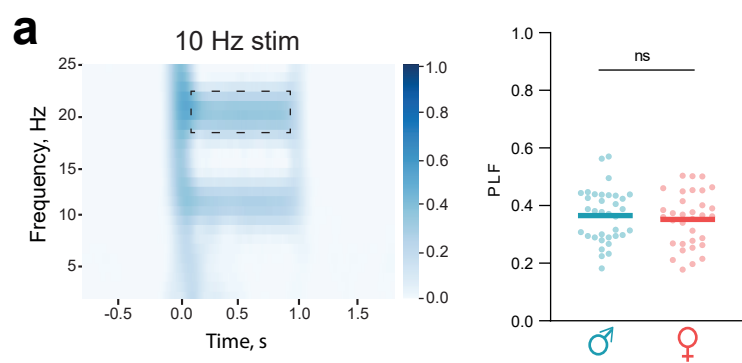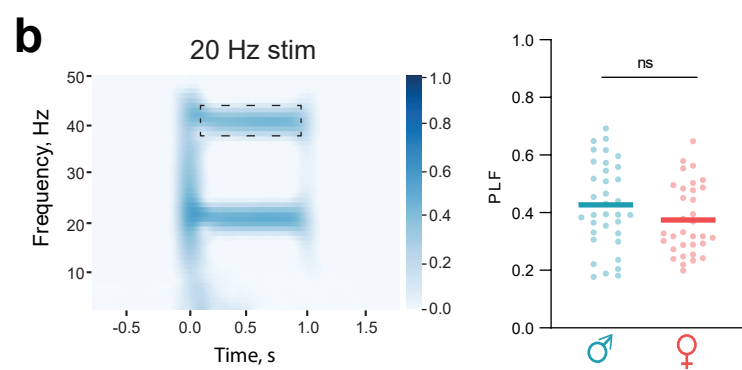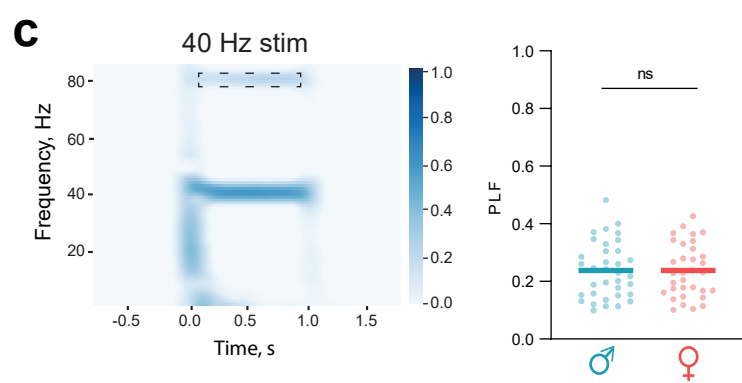

Supplement: Supplementary file 3 — Supplementary Material 3:Figure 3. Comparable harmonic response of ASSRs in male and female mice. a-c First harmonic responses of ASSRs at 10 (a), 20 (b) and 40 Hz (c) stimulation frequencies in male and female mice. Statistical comparisons were performed via the unpaired t test or the Mann-Whitney test. Significance notation: ns, non-significant (P > 0.05). Sample size: males, n = 35; females, n = 33. [file 13293_2025_773_MOESM3_ESM.pdf]

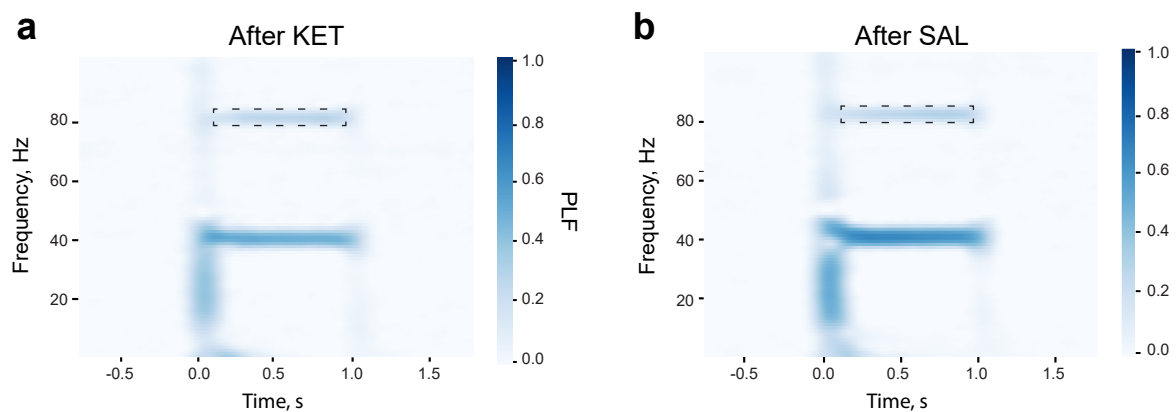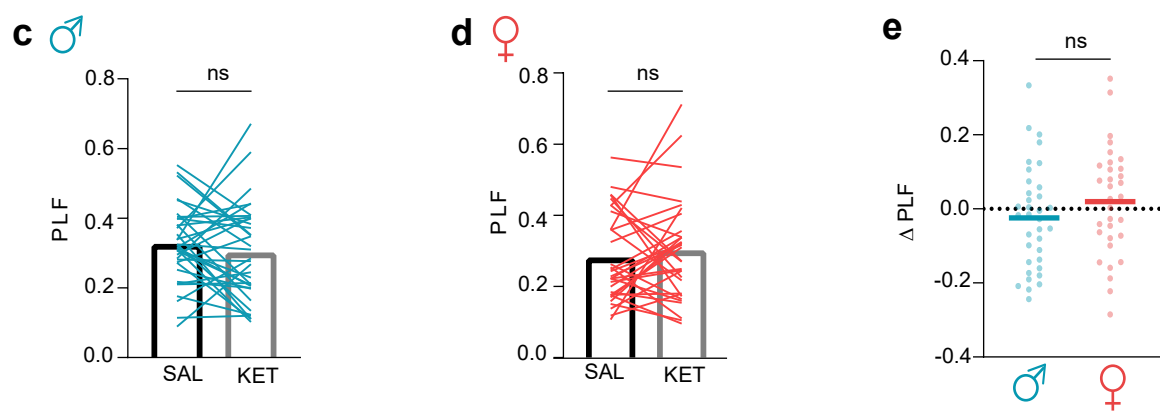

PLF

Supplement: Supplementary file 4 — Supplementary Material 4: Figure 4. Ketamine does not affect the 80 Hz harmonic response during 40 Hz stimulation in male and female mice. a, b Time‒frequency plots of the grand average phase-locking factor (PLF) following ketamine (KET, a) and saline (SAL, b) administration (n = 66). The black rectangles show the area of the first harmonics analysed. c, d PLF at the 80 Hz harmonic response during 40 Hz stimulation in male (c) and female (d) mice after KET and SAL. e Ketamine effects on the 80 Hz harmonic were calculated as Δ PLF (PLF_KET – PLF_SAL). Statistical comparisons of PLF values were performed via the paired t test or the Wilcoxon test; group comparisons of Δ PLF were evaluated using unpaired t test. Significance notation: ns, non-significant (P > 0.05). Sample size: males, n = 35; females, n = 31. [file 13293_2025_773_MOESM4_ESM.pdf]
